# From Corrosion Control to Cell Adhesion: Parascholzite as a Functional Interface for Biodegradable Zinc Alloys

**DOI:** 10.3390/ma19020416

**Published:** 2026-01-21

**Authors:** Jaroslav Fojt, Jakub Veselý, Jan Šťovíček, Jan Pokorný, Eva Jablonská, Zdeněk Míchal, Vojtěch Hybášek

**Affiliations:** 1 Department of Metals and Corrosion Engineering, Faculty of Chemical Technology, University of Chemistry and Technology, Technicka 5, 166 28 Prague, Czech Republic; veselbj@vscht.cz (J.V.); stovicen@vscht.cz (J.Š.); pokornyy@vscht.cz (J.P.); hybasekv@vscht.cz (V.H.); 2 Department of Biochemistry and Microbiology, Faculty of Food and Biochemical Technology, University of Chemistry and Technology, Technicka 5, 166 28 Prague, Czech Republic; jablonse@vscht.cz (E.J.); michald@vscht.cz (Z.M.)

**Keywords:** biodegradable zinc alloys, parascholzite coating, calcium phosphate surfaces, corrosion behavior, cell adhesion

## Abstract

Zinc-based alloys are promising candidates for biodegradable implant applications; however, their rapid initial corrosion and limited cytocompatibility remain major challenges. In this study, a Zn-Ca-P layer in a form of parascholzite (CaZn_2_(PO_4_)_2_·2H_2_O) was prepared on a Zn-0.8Mg-0.2Sr alloy via anodic oxidation followed by short-time biomimetic calcium–phosphate deposition. The formation mechanism, corrosion behaviour, and preliminary biological response of the modified surface were systematically investigated. The Zn-Ca-P layer formed a compact and crystalline phosphate layer that significantly altered the corrosion response of the zinc substrate in Leibovitz L-15 medium containing foetal bovine serum. Electrochemical measurements revealed a pronounced improvement in corrosion resistance and a transition from rapid active dissolution to a controlled, ion-exchange-driven degradation mechanism. The moderate solubility of parascholzite enabled the gradual release of Zn^2+^ and Ca^2+^ ions while maintaining surface stability during immersion. Preliminary cell adhesion experiments demonstrated a clear enhancement of cytocompatibility for the Zn-Ca-P-layer-coated samples, where cells readily adhered and spread, in contrast to the bare alloy surface, which showed lower cell attachment. The improved biological response is attributed to the phosphate-rich surface chemistry, favourable surface morphology, and moderated corrosion behaviour. Overall, the parascholzite-like layer provides an effective strategy with which to regulate both corrosion and early cell–material interactions of zinc-based biodegradable alloys, highlighting its potential for temporary biomedical implant applications.

## 1. Introduction

Recently, zinc and its alloys, together with other biodegradable materials based on magnesium or iron, have emerged as suitable alternatives to the metallic materials currently used in medicine (such as Ti-6Al-4V and Co-Cr alloys) [1,2]. Although these materials excel in terms of mechanical properties and corrosion resistance, in most cases, once the damaged tissue is healed, a secondary operation is required to remove the medical device. This increases both the financial costs and the risks that patients must undergo. The use of biodegradable materials, which degrade in a controlled manner directly in the patient’s body without adverse side effects after fulfilling their function, would eliminate these issues [2,3]. Of the biodegradable materials mentioned, zinc and its alloys currently appear to be the most promising, mainly due to their moderate corrosion rate and the absence of hydrogen evolution during corrosion processes, biocompatibility, and the essential role of zinc in human metabolism. For this study, a Zn-0.8Mg-0.2Sr alloy was used, which appears highly promising for bone repair, mainly due to its excellent combination of mechanical properties and acceptable degradation rate [2,4,5]. The corrosion behaviour of the Zn-0.8Mg-0.2Sr alloy was previously examined [1]. It was found that a layer containing ZnO, MgO, and MgCO_3_ naturally forms on the surface of the alloy, influencing the corrosion mechanism. Corrosion is typically initiated at the intermetallic phase boundaries due to micro-galvanic effects, with hydrozincite identified as one of the main corrosion products. The prevention of localised corrosion and simultaneous enhancement of uniform degradation and bioactivity can be achieved through surface treatments [6]. Various surface modification methods can be used to improve the properties of biodegradable zinc-based materials. The primary objective of most of these methods is to further improve the corrosion behaviour of the base material while enhancing its bioactivity and biocompatibility [1,2]. Surface treatment methods relevant to this study are described below. The first important group consists of electrochemical methods, such as anodic oxidation. In the case of zinc materials, this process results in the formation of a stable, homogeneous ZnO layer on the surface of the original material. By changing the parameters of anodic oxidation, it is possible to influence the thickness of the resulting layer, as well as the shape of the resulting nanostructure, such as nanowires [7], nanoplates [8,9], or nanotubes [10]. Among other things, these nanostructures contribute to reducing corrosion rates and improving antibacterial properties. Furthermore, it was reported that ZnO nanostructures promote Ca-phosphate precipitation, which can lead to the promotion of apatite formation [8,10]. Another group consists of biomimetic coatings, which are based on corrosion products formed during the degradation of zinc alloys in vivo corrosion, where the surface-adjacent layer composed predominantly of Zn, Ca, P, and O is naturally formed [11,12]. These involve exposing the sample to a supersaturated aqueous solution containing calcium and phosphate salts (e.g., SBF), which results in the formation of a protective layer rich in phosphorus and calcium, contributing to improved corrosion resistance and cell adhesion [2,13,14].

This article investigates methods for the preparation of a layer whose chemical composition corresponds to that of the naturally formed layer exhibiting good in vivo biocompatibility, modified to enhance cell adhesion and proliferation while avoiding the long-term suppression of biodegradability.

## 2. Experimental Section

### 2.1. Sample Preparation

In this paper, all measurements were carried out on Zn-0.8Mg-0.2Sr alloy samples.

The alloy was prepared from high-purity elemental metals (Zn: MTC Trading, s.r.o., Prague, Czech Republic, 99.995 wt.%; Mg: Magnesium Electron, Manchester, UK, 99.95 w.t%; Sr: Strem Chemicals, Newburyport, MA, USA, 99.9 wt.%) using an electric resistance furnace (EPS, Říčany, Czech Republic) at 550 °C without a protective atmosphere. Initially, the base metal was melted, after which alloying elements were added as pure metals. The melt was subsequently homogenised for 10 min and cast into a brass mould at room temperature. The resulting composition, determined via wet chemical analysis, corresponded to 99.02 wt.% Zn, 0.81 wt.% Mg, and 0.17 wt.% Sr.

Rectangular samples (20 × 20 × 8 mm) were prepared from the obtained ingot. For cellular assays, the blocks were subsequently cut into specimens with dimensions of 8 × 8 × 1.5 mm. The samples were mechanically ground using P600 abrasive SiC paper (ATM Qness GmbH, Mammelzen, Germany) before deposition. Subsequently, all samples were rinsed with distilled water and ethanol and dried with hot air. Samples were always rinsed again with distilled water, ethanol, and acetone and then allowed to air dry before proceeding further.

### 2.2. Preparation of ZnO Nanotubes

The preparation of the nanotubes (i.e., the anodic oxidation process) was carried out at room temperature in a solution consisting of sodium bicarbonate (0.6 g/L) and sodium dihydrogen carbonate (0.6 g/L) dissolved in distilled water. The nanotubes were prepared at 8 V for 7.5 min. Subsequently, the samples were carefully rinsed with distilled water and ethanol and dried in a stream of hot air.

### 2.3. Conversion of ZnO Nanotubes to a Zinc–Calcium–Phosphate Layer from Solution

The biomimetic conversion method was used for the deposition of the Zn-Ca-P layer on the already prepared nanotubes. Before immersion, the samples with nanotubes were briefly annealed at approximately 350 °C for 15 s to improve crystallinity and remove surface contaminants. Subsequently, the samples were immersed in a supersaturated calcium and phosphate solution at approximately 70 °C. The chemical composition of the solution is shown in Table 1. The samples were immersed in the solution for 25, 30, and 35 s and 2 h.

### 2.4. Surface Characterisation

The surface morphology of the samples after each deposition was analysed using a scanning electron microscope (SEM Tescan Vega 3, TESCAN, Brno, Czech Republic) with detectors of secondary electrons (SE) and backscattered electrons (BSE). Energy-dispersive spectroscopy (EDS, Oxford Instrument Aztec, Oxfordshire, UK) was used to evaluate the chemical composition of the prepared layers. The nanotubes’ parameters were evaluated from SEM pictures with ImageJ 1.54p software [15] from at least 20 measurements. XRD analysis was performed using a PANalytical X’Pert PRO instrument (Malvern Panalytical, Almelo, The Netherlands) (Bragg–Brentano geometry; Cu anode (λ = 1.5418); 40 kV; step size 0.0390; 2θ angle range 5–90°). Phase identification was performed using HighScore Plus 4.0 software (PANalytical) through comparisons with reference patterns from the ICDD PDF-4+ database.

### 2.5. Electrochemical Measurements

Electrochemical measurements were carried out with a Gamry Reference 600 potentiostat (Gamry Instruments, Inc., Philadelphia, PA, USA). Measurements were performed in a conventional three-electrode setup, using two graphite counter electrodes and a silver chloride reference electrode (SSCE, Gamry Instruments, Inc., Philadelphia, PA, USA). An exposed area of 0.5 cm^2^ was defined on the samples (working electrodes) placed in a holder with a top press fit using an O-ring (GUFERO Rubber Production s.r.o., Horní Třešňovec, Czech Republic). Leibovitz medium (L-15, Sigma, St. Louis, MO, USA) enriched with 2 mM glutamine and 10% FBS (foetal bovine serum, Sigma, St. Louis, MO, USA) at a constant temperature of 37 °C was used as electrolyte. For all samples, the polarisation resistance (R_p_) and open circuit potential (E_OC_) were monitored over a period of 7 days. For the Zn-Ca-P-coated samples, the electrolyte was changed after 7 days, and the measurements continued for an additional 2 days.

### 2.6. Initial Cell–Material Response

Sterilised materials (2 h in 70% EtOH and 2 h under UV, Prolux G^®^ GIP65 36 W, NEXA, Piešťany, Slovakia) with the Zn-Ca-P layer were placed into a 24-well plate, and 0.75 mL of L-15 medium +10% FBS + glutamine was added. Materials were preincubated for 24 h at 37 °C. The HFOB 1.19 cell line (ATCC, CRL-3602, Manassas, VA, USA) [16] was cultivated in DMEM/Hams medium (Sigma, D6434) with 10% FBS, glutamine, and G418 at 34 °C. For the experiment, cells were trypsinised, centrifuged (200× *g*, 5 min), and resuspended in L-15 medium + 10% FBS + glutamine. In total, 0.75 mL of cell suspension (concentration 2.5 × 10^5^ cells/mL) was added onto the materials in each well (the seeding density was 100,000 cells/cm^2^). Cells were incubated at 37 °C for 24 h. Material without a treatment (Zn-0.8Mg-0.2Sr bare alloy) was used as a control material.

For SEM, the medium was removed, cells were washed with PBS, and materials were transferred to new wells. Cells were fixed as in [17] using Karnovsky’s solution for 1.5 h at room temperature. The samples were then washed twice with 0.1 M cacodylate buffer. Cells fixed on the samples were dehydrated through a 10 min incubation in increasing concentrations of EtOH (50%, 70%, 90%), followed by a wash in absolute EtOH and a final incubation in absolute EtOH for 5 min. The samples with cells were then transferred to acetone and dried using CPD (Leica EM CPD300, Leica Microsystems GmbH, Wetzlar, Germany). Finally, the samples with cells were sputter-coated with 10 nm of gold (Leica EM ACE600).

For cell metabolic activity assessment, after the incubation, materials were removed from the wells, cells originally growing in the vicinity of the material were washed, and resazurin in PBS was added (final concentration: 25 μg/mL). After 2 h of incubation, fluorescence was measured (ex/em 544/590 nm) using Fluoroskan Ascent FL (Ascent 2.6 Software). Metabolic activity was expressed relatively to the cells growing on the bottom of the well without any material.

## 3. Results and Discussion

### 3.1. Preparation of the Biomimetic Layers

**Figure 1:**
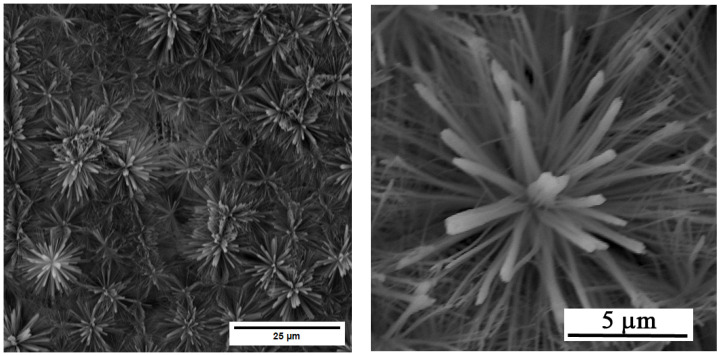
SEM image of the surface layer of ZnO nanotubes.

A homogeneous layer of ZnO nanotubes with flower-like (Figure 1) morphology was successfully prepared using the optimised anodization parameters from the literature [10]. The tube length varied from 4 to 7 μm, and the outer diameter was 87 nm with a standard deviation of 31 nm.

**Figure 2:**
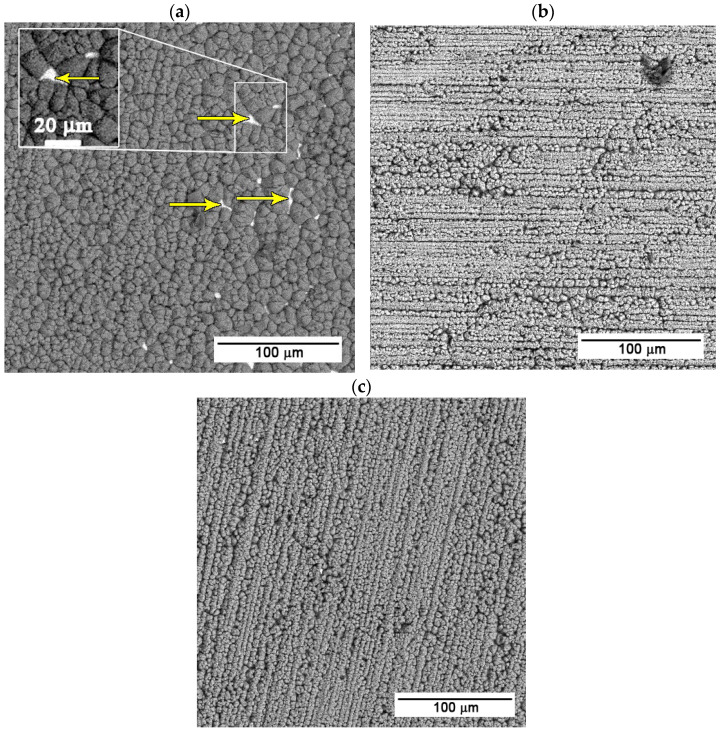
SEM (BSE) images of samples with Zn-Ca-P layer with exposure times of (**a**) 25 (arrows pointing to the layer defects), (**b**) 30, and (**c**) 35 s.

The zinc–calcium–phosphate layer was prepared after the preparation of the ZnO nanotube layer through the immersion of the samples in the supersaturated solution of Ca(NO_3_)_2_ and KH_2_PO_4_ at 70 °C. The samples were exposed to the solution for 25, 30, and 35 s. It can be seen in the SEM (BSE) images (Figure 2) below that with increasing exposure time, the surface coverage of the samples becomes more uniform.

As can be seen in Figure 2a, the shortest exposure time, i.e., 25 s, results in incomplete surface coverage, with clearly visible porosity in several areas of the prepared layer. These surface defects represent potential weak points that can negatively affect the protective function of the layer and reduce its overall effectiveness.

**Figure 3:**
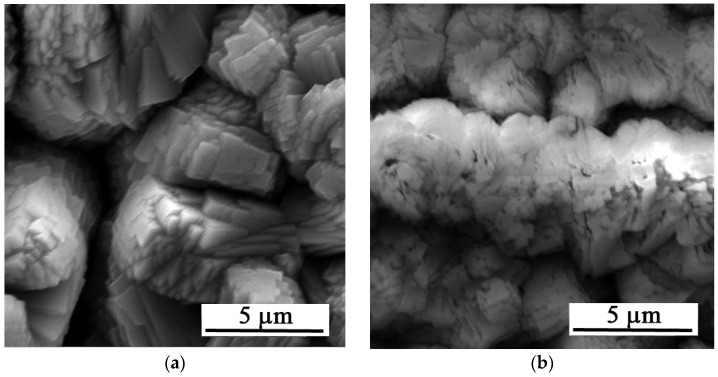

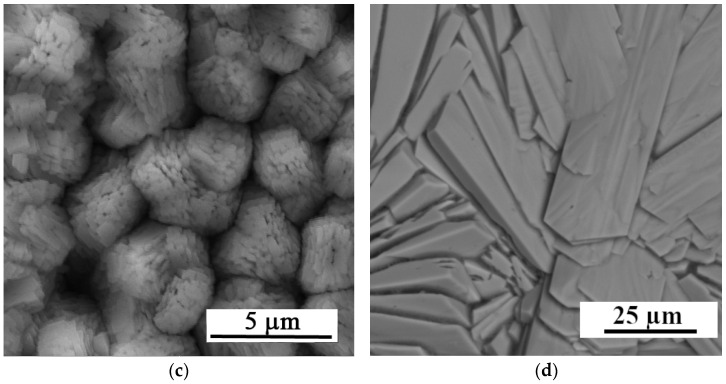
Detailed SEM images of the Zn-Ca-P layers with exposure times of (**a**) 25, (**b**) 30, (**c**) 35 s, and (**d**) 2 h.

Figure 2b,c present the sample surfaces after immersion for 30 and 35 s, respectively. In both cases, a more homogeneous and compact Ca-P layer without significant surface defects is formed compared to the shorter exposure time. For the sample exposed for 35 s (Figure 2c), the prepared layer is more compact and uniform than the layer on the sample exposed for 30 s (Figure 2b). Detailed images of the deposited layers are in Figure 3.

**Figure 4:**
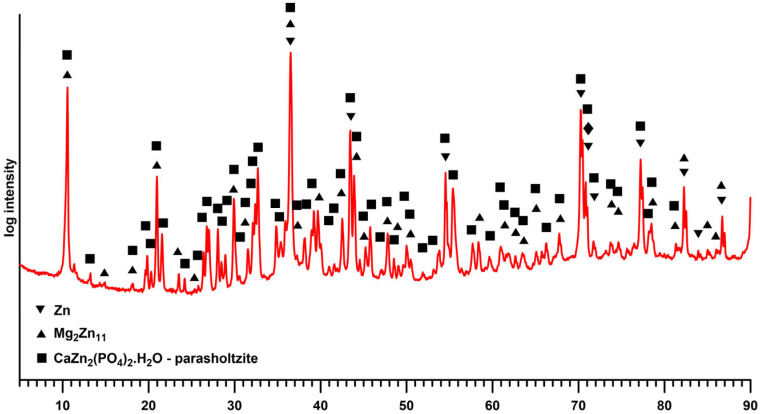
XRD spectrum of the sample with a Zn-Ca-P layer prepared with an exposure time of 2 h.

Considering the defects of the surface layer prepared for 25 s, only the samples with 30 and 35 s exposure times were selected for further testing. Given the satisfactory quality of the surface layers produced, longer exposure times were not studied, with the exception of XRD analysis, where the thicker layer prepared at a conversion time of 2 h provided a more unambiguous signal. The XRD analysis (Figure 4) identified the layer as parascholzite, which is consistent with the EDS analysis across all conversion times.

### 3.2. Electrochemical Measurements

**Figure 5:**
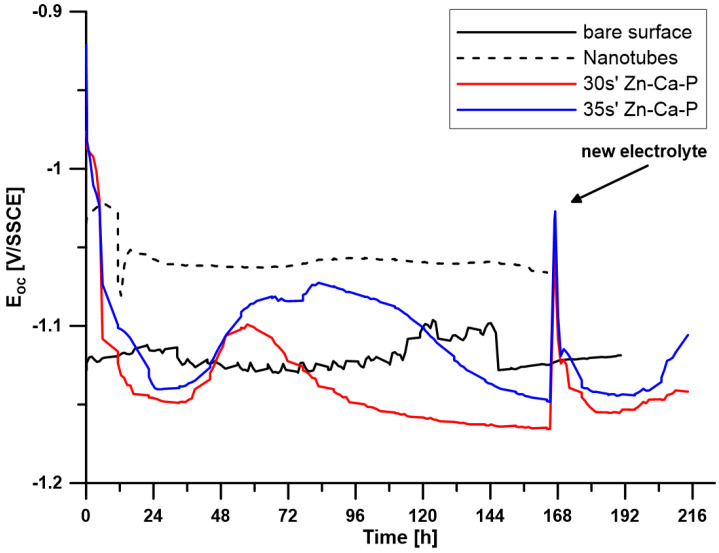
Time-dependent changes in E_OC_ for all tested samples (30 s′ and 35 s′ denote the time in the conversion bath).

**Figure 6:**
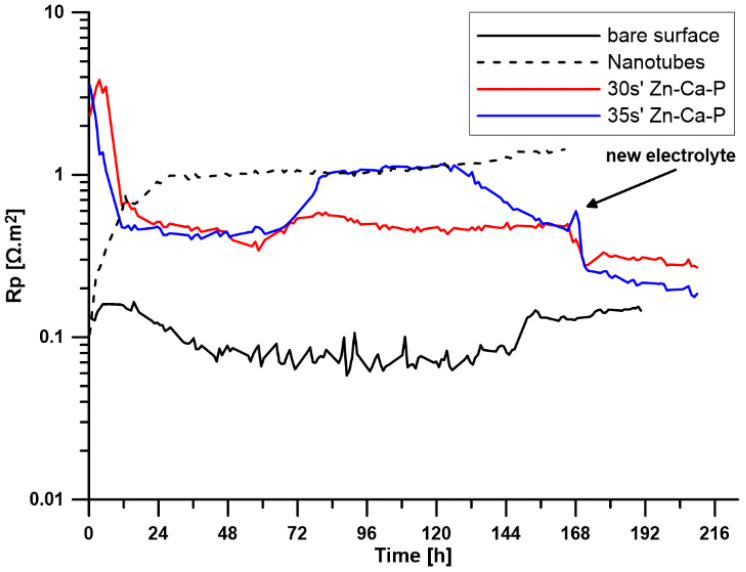
Time-dependent changes in R_p_ for all tested samples (30 s′ and 35 s′ denote the time in the conversion bath).

To investigate the corrosion behaviour of the prepared surface layers, the open circuit potential (E_OC_) and polarisation resistance (R_p_) were measured over a period of 7 days. For samples with a Zn-Ca-P layer (30 s, 35 s), the electrolyte was replaced after 7 days, and the measurements continued for another two days. The time-dependent changes in E_OC_ values are shown in Figure 5, and the changes in R_p_ values are shown in Figure 6.

Figure 5 indicates that the reference sample made of pure zinc maintains a relatively stable open circuit potential in the range of −1.10 to −1.12 V throughout the exposure period, suggesting relatively stable behaviour. The polarisation resistance varied between 0.07 and 0.2 Ω·m^2^.

The sample with a surface modified with ZnO nanotubes shows a stable course of both E_OC_ with values around −1.05 V and R_p_ with values around approximately 1 Ω·m^2^ throughout the exposure period. This indicates high stability of the prepared layer during exposure and better corrosion resistance compared to pure zinc.

For both samples with a Zn-Ca-P layer, after the initial stabilisation in the first 48 h, there is a shift towards more positive potential values. For the layer with a deposition time of 35 s, this increase is more significant compared to the deposition time of 30 s and remains at more positive values until the electrolyte is replaced after 168 h of exposure. After the electrolyte was replaced, there was an increase in potential values and subsequent stabilisation at a level close to the values before the electrolyte was replaced.

Another difference can be observed in the R_p_ curve. The sample with a layer deposited for 30 s exhibited an almost constant polarisation resistance value throughout the exposure period, while the layer deposited for 35 s showed an increase in R_p_ values after approximately 72 h, at which it remained stable for the next 50 h and then decreased slightly to values identical to those of the second sample. This trend indicates that the layer formed by deposition for 35 s is more effective in suppressing corrosion processes in the initial phase, but after a certain period of time, it begins to degrade in a controlled manner.

**Figure 7:**
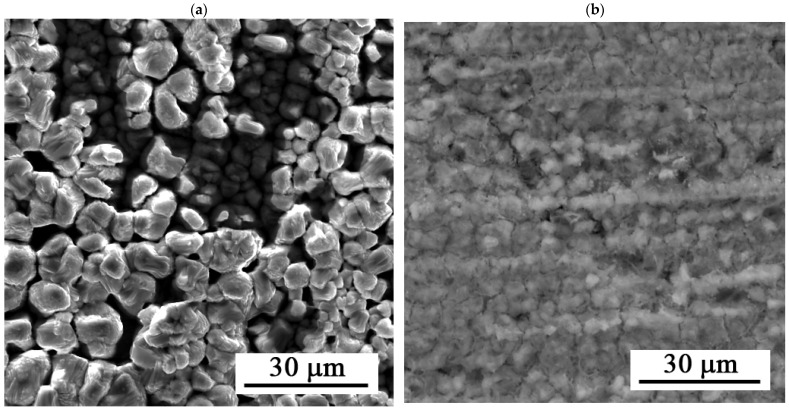
SEM images of the Zn-Ca-P after 216 h of exposure: (**a**) 30 s and (**b**) 35 s.

Figure 7 shows the surface of the layers after 216 h of exposure. The figure shows uniform dissolution of the layer.

### 3.3. Initial Cell–Material Response

**Figure 8:**
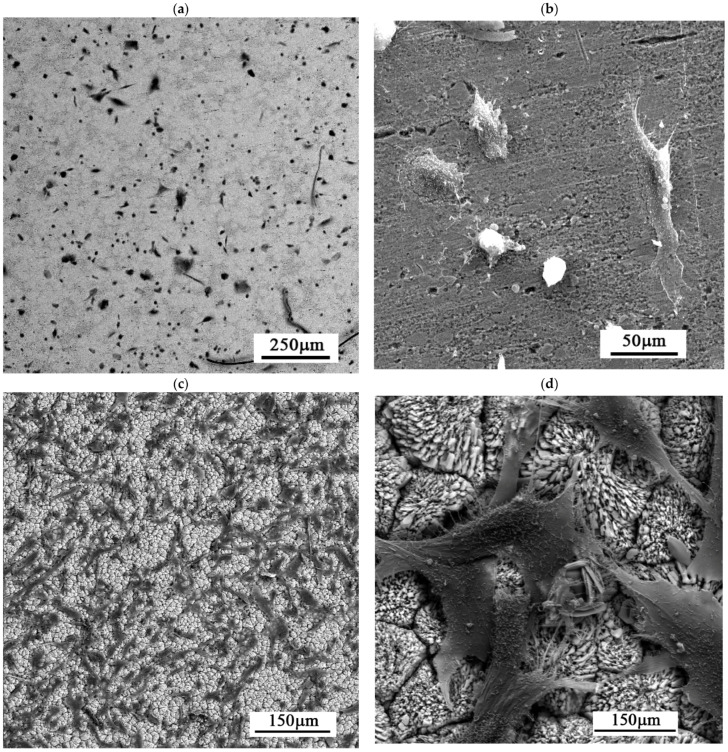

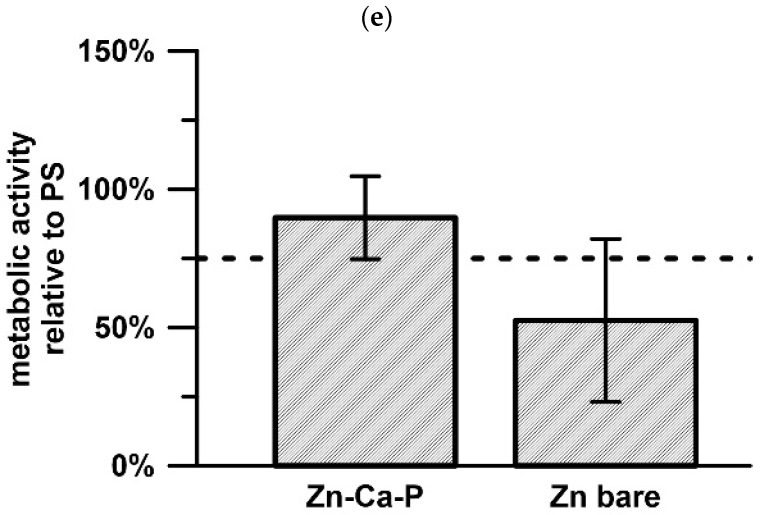
HFOB 1.19 cells on (**a**,**b**) bare surface and (**c**,**d**) Zn-Ca-P layer; (**e**) relative metabolic activity after cultivation in the vicinity of the material.

Cell adhesion on the investigated surfaces was evaluated qualitatively using microscopy after incubation directly on the surface. Cells were clearly observed on the Zn-Ca-P coated samples, where they adhered to the surface and exhibited a spread morphology. In contrast, the uncoated alloy substrate showed a lower level of cell coverage of the surface, with the surface remaining partially free of cellular coverage (Figure 8a,b). On the Zn-Ca-P layer, cells were distributed across the surface and formed visible attachment points, indicating successful initial adhesion (Figure 8c,d). Furthermore, a second experiment was conducted, representing an equivalent of the extract test but performed under more physiologically relevant conditions, to assess the metabolic activity of osteoblasts cultured directly in the vicinity of the material (Figure 8e). Compared to the untreated surface, the objective was achieved, as evidenced by a reduced data scatter and an increased median value of viability.

## 4. Discussion

The surface modification of the Zn-0.8Mg-0.2Sr alloy via anodic oxidation followed by biomimetic calcium–phosphate deposition produced a bilayer coating with markedly improved corrosion resistance and controlled degradation in the physiological L-15 medium containing foetal bovine serum (FBS). Structural characterisation confirmed that the deposited Zn-Ca-P layer corresponded to parascholzite (CaZn_2_(PO_4_)_2_·2H_2_O), a zinc-containing phosphate phase with moderate solubility and excellent interfacial compatibility. The formation of this phase and its corrosion performance can be understood by linking the electrochemical data with the physicochemical processes governing oxide growth, phosphate precipitation, and ion-mediated degradation in complex biological media.

The anodic oxidation step at 8 V for 7.5 min in a NaHCO_3_/NaH_2_CO_3_ electrolyte produced a flower-like ZnO nanotubular morphology. The underlying mechanism involves field-assisted oxidation and dissolution, where localised electric fields promote pore initiation and self-organised nanotube propagation [9,10]. The short annealing treatment at 350 °C enhanced the crystallinity and mechanical stability of the ZnO layer. The resulting nanostructured ZnO surface exhibits high roughness, defect density, and abundant surface hydroxylation, which increase surface reactivity and provide favourable sites for ion adsorption and nucleation during the subsequent phosphate treatment [2,7].

During immersion in the supersaturated Ca-P solution at 70 °C, the ZnO nanotubes facilitated the heterogeneous nucleation of phosphate phases. Also, due to the dissolution of ZnO, Zn^2+^ ions were introduced into the solution and incorporated into the growing calcium–phosphate lattice, forming parascholzite (CaZn_2_(PO_4_)_2_·2H_2_O) as the dominant phase. The nucleation of parascholzite proceeds through a Zn-mediated pathway, where the local Zn^2+^ concentration suppresses the formation of Ca-rich apatite and stabilises the mixed Ca-Zn phosphate [3,6,18]. The morphology of layer evolved with immersion time: short immersion (25 s) yielded discontinuous crystallites, while 30–35 s resulted in the formation of a compact layer composed of an interconnected globular substructure with globule diameters in the range of 10–25 µm, while the individual globules are formed by submicrometer-sized crystals oriented along the nanotubular substrate. This transition reflects the gradual densification and crystallisation process characteristic of phosphate coatings formed under kinetic control [19]. The structural compatibility between parascholzite and ZnO ensures strong interfacial bonding and minimises the risk of interfacial degradation during immersion.

Electrochemical measurements confirmed that both the ZnO and Zn-Ca-P layers significantly improved corrosion resistance in L-15 + FBS medium. The uncoated Zn-0.8Mg-0.2Sr alloy exhibited an open circuit potential (EOC) of approximately −1.11 V and a low polarisation resistance (R_p_ ≈ 0.2 Ω·m^2^), reflecting rapid active dissolution. The ZnO-coated samples showed a positive shift to −1.05 V and R_p_ ≈ 1 Ω·m^2^, remaining stable throughout seven days of immersion. The Zn-Ca-P-coated samples displayed the most pronounced improvement: the 30 s layer maintained an EOC near −1.00 V and R_p_ ≈ 1 Ω·m^2^, while the 35 s layer reached an EOC ≈ −0.95 V and R_p_ up to 1.5 Ω·m^2^ after 72 h, remaining stable until 168 h. These findings indicate that the crystalline Zn-Ca-P layer acted as an efficient barrier, restricting ionic transport and reducing anodic activity during the initial immersion period.

The corrosion mechanism in L-15 + FBS medium involves several overlapping stages. Initially (0–48 h), the Zn-Ca-P layer functions as a compact barrier preventing electrolyte penetration. The parascholzite phase, being sparingly soluble, resists chloride-induced breakdown while maintaining ionic exchange with the surrounding solution. Proteins from FBS rapidly adsorb onto the hydrophilic phosphate surface, forming a transient organic film that further reduces electrochemical activity [20]. In the subsequent stage (48–168 h), chloride ions gradually diffuse through micro-porous regions and interact with the outer phosphate layer, leading to slow surface hydration and limited ion exchange. Controlled dissolution of the Zn-Ca-P layer releases Zn^2+^ and Ca^2+^ ions, which react with phosphate and carbonate species in the medium to precipitate secondary corrosion products such as Zn_3_(PO_4_)_2_·4H_2_O and hydrozincite (Zn_5_(CO_3_)_2_(OH)_6_) [5]. These products contribute to local pH buffering and further passivation.

Micro-galvanic effects between the α-Zn matrix and intermetallic particles such as Mg_2_Zn_11_ and SrZn_13_ remain the main source of localised anodic activity [1,5]. However, the presence of the Zn-Ca-P layer mitigates these effects by homogenising the electrochemical surface potential and reducing the exposure of intermetallics to the solution. With over-extended immersion (>168 h), the system enters a quasi-steady state characterised by slow and uniform degradation. The partial transformation of parascholzite into hydrated zinc phosphates and Zn(OH)_2_ maintains corrosion control without abrupt coating failure. The gradual decrease in R_p_ observed after 168 h thus represents a controlled transition from barrier-dominated to ion-exchange-controlled corrosion—a desirable feature for biodegradable materials [3,18].

The corrosion potentials and resistances achieved here compare favourably with those in literature data. Dong et al. [10] reported R_p_ ≈ 0.8 Ω·m^2^ for ZnO nanotubes in simulated body fluids, whereas the ZnO/Zn-Ca-P bilayer reached up to 1.5 Ω·m^2^ with a 150 mV positive potential shift. Similar improvements have been reported for ZnO/Ca-P systems designed for biodegradable applications [2,6]. The enhanced stability of parascholzite relative to hydroxyapatite in chloride-rich environments accounts for the prolonged protection observed. Its intermediate solubility ensures a gradual ion release, while its structural incorporation of Zn^2+^ promotes interfacial coherence and prevents delamination. The Zn-Ca-P layer thus provides both early-stage protection and long-term controlled degradation.

Considering the described protective effect and the associated coherence of the layer with the substrate, which is intentionally limited primarily to early-stage interactions, the performed cell-based tests focused mainly on the initial cellular response, particularly cytotoxicity in the vicinity of the material and primary cell adhesion. Non-cancerous hFOB 1.19 osteoblasts were therefore selected to ensure physiologically normal behaviour, unaffected by oncogenic alterations, together with their increased sensitivity to Zn^2+^ ions. Unlike the commonly used MG-63 and U-2OS cell lines, the hFOB 1.19 (human foetal osteoblast) cell line is non-cancerous and conditionally immortalised and exhibits characteristics more closely resembling those of osteoblasts. Based on the preliminary cell-based evaluations, the markedly different cell adhesion behaviour observed on the Zn-Ca-P coated and uncoated alloy surfaces highlights the critical role of surface modification in regulating the biological performance of zinc-based materials. Bare zinc surfaces are known to undergo rapid initial corrosion in physiological environments, which can result in locally elevated Zn^2+^ concentrations and abrupt changes in interfacial pH. Such conditions adversely affect protein adsorption and inhibit the formation of stable focal adhesions, leading to poor cell attachment during the early stages of exposure [5,21,22].

The presence of the Zn-Ca-P layer significantly alters this interfacial environment. As a zinc-containing calcium phosphate, parascholzite acts as a chemically stable and moderately soluble surface layer that moderates corrosion and limits direct contact between cells and the metallic substrate. Calcium phosphate surfaces are well recognised for their strong affinity toward serum proteins, including fibronectin and vitronectin, which play a key role in integrin-mediated cell adhesion and spreading [19,23]. The adsorption of these proteins onto the Zn-Ca-P layer surface likely leads to the formation of a bioactive interphase that promotes cell anchorage. Surface morphology further contributes to the observed biological response. The crystalline parascholzite layer exhibits micro- to nanoscale features that increase surface roughness and available contact area, which has been shown to enhance cytoskeletal organisation and focal adhesion formation [2,24,25]. Hierarchical surface architectures combining chemical bioactivity with topographical cues have been repeatedly demonstrated to improve early cell–material interactions, even in the absence of additional biochemical functionalization. In addition to topography and protein adsorption, ionic interactions are likely to play an important role. Controlled release of Zn^2+^ and Ca^2+^ ions from parascholzite may contribute to a favourable biological microenvironment [18,26]. While excessive zinc ion concentrations are cytotoxic, low and sustained Zn^2+^ levels have been reported to support cell proliferation and stimulate osteogenic activity [27]. Similarly, calcium ions participate in cell signalling pathways associated with adhesion and differentiation. The corrosion mechanism of parascholzite—characterised by gradual ion exchange rather than rapid dissolution—thus appears compatible with early cellular activity. Taken together, these factors explain why cells readily adhered to the Zn-Ca-P layer coated surface, while attachment to the bare Zn substrate was lower. The results underscore the importance of phosphate-based surface layers in decoupling corrosion kinetics from biological response in biodegradable zinc systems. Similar improvements in cytocompatibility have been reported for zinc alloys modified with calcium phosphate or phosphate-containing coatings, confirming that such surface modifications are an effective strategy for enabling early cell–material interactions [2,5,22,25]. The discussed effects were also reflected, to a limited extent, in the relative metabolic activity of osteoblasts in the immediate vicinity of the material, where an improvement was observed compared to the bare alloy.

Despite the limitations associated with short-term in vitro cell-based testing, the results offer valuable information on the initial cell–material interactions. In this context, further investigations are warranted to verify the expected osseoinductive potential of the layer degradation products, particularly under long-term in vitro and in vivo conditions.

## 5. Conclusions

This study demonstrates that a layer based on Zn-Ca-P in the form of parascholzite (CaZn_2_(PO_4_)_2_·2H_2_O) provides an effective and multifunctional surface modification for Zn–Mg–Sr biodegradable alloys. The layer forms a compact, crystalline phosphate interface that fundamentally alters the corrosion behaviour of zinc, transforming rapid initial dissolution into a controlled, ion-exchange-driven degradation process compatible with physiological environments. Rather than acting as a passive protective film, the Zn-Ca-P layer dynamically interacts with the surrounding medium, enabling gradual transformation while maintaining surface stability. Importantly, this controlled corrosion response directly translates into an improved biological performance. Preliminary cell adhesion experiments revealed that cells readily adhere and spread on the Zn-Ca-P-layer-covered surface, whereas the uncoated zinc substrate remains unfavourable for early cell attachment. The enhanced cytocompatibility is attributed to the phosphate-rich surface chemistry, favourable surface morphology, and moderated release of zinc and calcium ions, which together create a biologically supportive interfacial environment. By decoupling corrosion kinetics from early biological response, the Zn-Ca-P layer enables zinc-based alloys to simultaneously fulfil the key requirements of biodegradable implants: initial stability, predictable degradation, and compatibility with cellular attachment. These findings highlight the submicrostructured parascholzite-like layer as a promising phosphate interface for guiding both material degradation and early biointegration.

## Figures and Tables

**Table 1:** Composition of the solution for the preparation of the Ca-P layer.

Substance	Molar Concentration [mol·L^−1^]
Ca(NO_3_)_2_	1.46
KH_2_PO_4_	0.87

## Data Availability

The original contributions presented in this study are included in the article. Further inquiries can be directed to the corresponding author.
